# Anatomo-pathological aspects of pelvic organ prolapse: analytical study of round and utero-sacral ligaments of Congolese women during the mass campaign in two hospitals of city of Kananga

**DOI:** 10.11604/pamj.2025.50.26.45534

**Published:** 2025-01-14

**Authors:** Antoine Tshimbundu Kayembe, Patrick Kahindo Muyayalo, Andy Mbangama Muela, Rahma Raschid Tozin

**Affiliations:** 1 Department of Gynaecology and Obstetrics, Faculty of Medicine, University Notre-Dame of Kasayi, Central Kasaï, Democratic Republic of Congo,; 2 Department of Gynaecology and Obstetrics, Faculty of Medicine, University of Kinshasa, Kinshasa, Democratic Republic of Congo

**Keywords:** Anatomo-pathological aspects, pelvic organ prolapse, Bon-Berger Hospital, Saint-Georges Hospital, Kananga

## Abstract

**Introduction:**

pelvic organ prolapse is the falling into or out of the vaginal cavity of the uterus or rectum or bladder lined with vaginal walls due to several lesions of the ligaments and fascia of the pelvic floor marked by their weakening or hypotonia. The objective of our present study is to determine the anatomo-pathological aspects associated with pelvic organ prolapse during the surgical care campaign in the hospitals of Bon-Berger and of Saint-Georges of the city of Kananga in the Democratic Republic of Congo.

**Methods:**

this is an analytical study based on the morphological anatomo-pathological examination of the round and uterosacral ligaments of 100 consenting patients divided into two groups with and without pelvic organ prolapse treated in the Gynecology Departments of Bon-Berger hospitals in Tshikaji and Saint-Georges in Katoka in the city of Kananga, from January 1^st^ to July 31^st^, 2023. Non-probability convenience sampling helped in the selection of cases. The Anova test and the Chi test are used in statistical analyses.

**Results:**

the average age of our patients with pelvic organ prolapse was 57.18 (SD: 8.17) years and their average parity was 7.76 (SD: 1.04) delivery. This average parity was significantly increased compared to those of patients without prolapse. Fibrosis was present in 92% of cases of pelvic organ prolapses, congestion in 62%, inflammatory infiltrate in 54%, and smooth muscle in 22% of cases; the comparison of patients with pelvic organ prolapse to those without prolapse did not find statistically significant differences between these two groups concerning the presence of fibrosis, congestion, the inflammatory infiltrate, and smooth muscle.

**Conclusion:**

pelvic organ prolapse is a fibrotic and inflammatory disease not significant and the search for inflammatory and fibrotic markers is essential in our city of Kananga in Democratic Republic of Congo.

## Introduction

Pelvic organ prolapse (POP) is the falling into or out of the vaginal cavity of the uterus or rectum or bladder lined with vaginal walls due to several lesions of the ligaments and fascia of the pelvic floor marked by their weakening or hypotonia [1,2]. The predominant clinical manifestations are the anterior and or posterior colpocele as well as hysterocele [1,2]. Although POP is non-fatal, it causes significant consequences on the quality of life of patients who are affected by multiple serious social and psychological problems such as the loss of self-esteem [3-7].

According to the World Health Organization (WHO), POP is a real health problem affecting approximately 50% of women who have given birth, and its lifetime prevalence is estimated from 20 to 50%. It is one of the major reasons for gynecologic surgery in aging women [3-6,8]. Several studies have reported the lifetime risk for a woman to undergo at least one surgery for POP and urinary incontinence of 11%, with a 10-year reoperation rate of 17% [3,9,10]. The global prevalence of POP has been estimated to be approximately 9%, and however, this figure is evaluated to be closer to 20% in low-income countries [11]. In Africa, multiple studies conducted in sub-Saharan countries such as Gambia, Ghana, Tanzania, and Ethiopia have found prevalences varying from 12 to 65% [12-15]. The prevalence of POP is 24% with of its recurrence of 8.69% in the town of Kananga in the Democratic Republic of Congo [16]. The factors associated with POP specific to the city of Kananga in the Democratic Republic of Congo are menopause, vaginal delivery, multiparity, obstetric trauma, fetal macrosomia malnutrition (BMI less than 18.5 Kg/m^2^), and intense physical work [17] whereas in Kinshasa, these factors are represented by obstetric trauma, obesity, vaginal delivery, menopause, and fetal macrosomia [18].

The pelvic organs are supported by the pelvic floor muscles, the bony pelvis, the pelvic ligaments, and the pelvic fascia. Structural, biochemical, and functional abnormalities in pelvic connective tissues have been shown to contribute to the genesis of POP [19,20]. On the anatomo-pathological level, several studies conducted worldwide have reported the presence of inflammatory infiltrate, engorged capillary vessels, smooth muscle, and fibrosis in the connective tissues of prolapsed pelvic organs. These anatomo-pathological aspects determine the types of biochemical anomalies including the secretion of inflammatory mediators such as interleukins, matrix metalloproteinases…; and functional anomalies responsible for the weakening of the pelvic ligaments at the base of the outbreak of POP. These aspects are associated with POP and their presence implies those of these anomalies listed above [20,27].

The lack of data concerning the anatomo-pathological aspects associated with POP in our city of Kananga justifies this present study whose objective is to determine the anatomo-pathological aspects associated with POP in round and utero-sacral ligaments of Congolese women registered during the mass campaign in two hospitals of Bon-Berger and Saint-Georges of the city of Kananga.

## Methods

**Study design and setting:** this is an analytical study of morphological anatomo-pathological examinations of round and uterosacral ligaments in patients with POP constituting the study group compared to those in patients without prolapse (i.e. patients who suffer from other benign gynecological diseases) constituting the comparison, all having undergone total hysterectomy recorded during the mass campaign which was organized in the gynecological departments of two hospitals of the city of Kananga: Bon-Berger Hospitals and Saint Georges, from January 1^st^ to July 31^st^, 2023. These two hospitals were chosen because of the presence of trained and experienced medical staff, the high attendance of patients who suffer from POP, and the more or less free management of POP through the various surgical care campaigns in the fistula cure account. Therefore, these two hospitals constitute references for the management of POP in our city of Kananga.

**Study population:** the population of our study consists of patients who have signed the informed consent form, aged between 40 and 79 years, who suffer from POP for the study group and other benign gynecological pathologies for the comparison group and who were treated during the mass campaign in the gynecology departments of the Bon-berger hospitals and Saint Georges in city of Kananga from January 1^st^ to July 31^st^, 2023, and matched according to age plus or minus 3 years. We used the non-probabilistic sampling of convenience for case selection. The sample size was estimated thanks to the limitations of our study in time and space. The criteria cited below have allowed us to include patients in this study: patients who signed the informed consent form, aged between 40 and 79 years who suffered from POP (study group) and other benign gynecological diseases (comparison group), and who underwent total hysterectomy during the surgical care campaign in the gynecology departments of the Bon-berger hospitals and Saint-Georges in city of Kananga from January 1^st^ to July 31^st^, 2023. We excluded all patients who refused to sign the informed consent form, those who had already undergone surgery for POP, those suffering from malignant gynecological pathologies, and those on hormone replacement therapy in case of complicated menopause. Our sample size was calculated using the following formula [17,18,24]:


$$n\geq \frac{\left(1+\frac{1}{c}\right){\left(Z_{\alpha }+Z_{1-\beta }\right)}^{2}p\left(1-p\right)}{{\left(p^{0}-p^{1}\right)}^{2}}$$

Where: n = number of cases; c = number of cases in the comparison group by study case; P^0^= expected proportion of exposed cases in the comparison group (0.0116); P^1^= expected proportion of exposed cases in the study group (0.105).


$$p_{1}=\frac{p_{0}xOR}{1+p_{0}\left(OR-1\right)}$$

p = proportion of exposed subjects in both groups (study and comparison) (0.058):


$$p=\frac{p_{1}+cp_{0}}{1+c}$$

Z_α_= Z value for the first type risk (1.645); α = the risk of type I error (0.05); Z_1-β_= Z value corresponding to a surface equal to the power of the test (1 - β). The latter constitutes the probability of finding a significant difference (1.282), (1-β) = the desired power (0.9) [17,18,24]. OR = minimum OR that is set for the study to be of public health interest and estimated at 20 on the basis of studies on the Visco and Yuan model [28]. The calculated sample size is greater than 47 cases and we increased it to 50 cases for the study group and 50 cases for the comparison group. The number of cases in the comparison group is equal to the product n x c. A comparison case will be matched to a study case (c = 1) and the matching criterion is the age of patients plus or minus 3 years.

**Data collection:** we collected the data from the interview of patients and the search in the medical records of patients and the registers of the gynecology services of two hospitals and noted in the data collection record. We used the following variables of study: age of patients, parity, diagnosis, types of tissues sampled, and nature of anatomo-pathological lesions. Our data were collected in the following manner: after the written and signed consent for each patient admitted to this study, the collection of information was done by interviewing patients and searching in the medical records. Two tissue biopsies of 10 mm in length each are taken from each woman during surgery: one from the uterosacral ligaments and one from the round ligament. All biopsies were fixed in 10% formalin and preserved until the time of morphological anatomo-pathological analyses.

**Morphological staining:** all our tissues collected were systematically fixed in 10% buffered formalin and transformed into paraffin blocks by routine methods. The sections of the samples at 5 µm thickness were made using the microtome, mounted on coated slides and stained using the standard staining technique with hematoxylin and eosin whose protocol is established in the anatomo-pathological laboratory of the University Clinics of Kinshasa.

**Morphological staining:** we deparaffinized the sections by bathing them in xylene for 10 to 20 minutes. Filter the hematoxylin. We rehydrated the sections by immersing them in descending alcohols: 100% alcohol for 1 to 2 minutes, 95% alcohol for 1 to 2 minutes, and 70% alcohol for 1 to 2 minutes. We rinsed them with tap water, then with distilled water before staining them with hematoxylin for 3 to 5 minutes. We rinsed the sections with tap water for 10 seconds and bathed them in hydrochloric alcohol (1% HCl in 70% alcohol) for 5 minutes for their differentiation. We rinsed the slides with running water for 15 minutes before staining them with eosin for 1 to 4 minutes. We proceeded to the dehydration and differentiation of slides/sections by dipping them 5 to 6 times successively in ascending alcohols: 95% alcohol for 3 minutes, then 100% alcohol for 3 minutes. We rinsed the slides in xylene for 5 minutes before mounting these slides with a mounting medium (Permount or DPX). The sections were covered with Canada balsam and coverslip with bubble removal and drying for 1 hour and mounted on the microscope for examination at 200-400X magnification.

**Figure 1:**
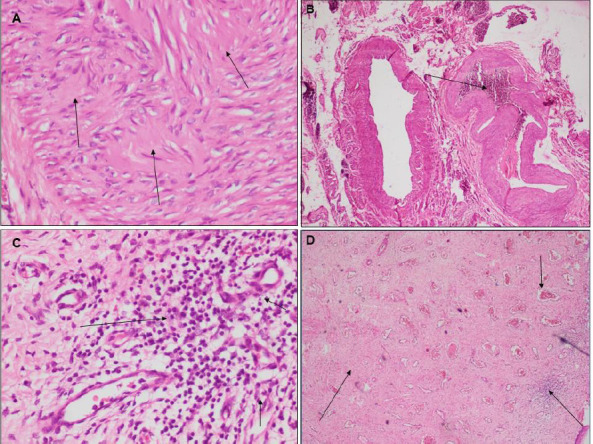
anatomo-pathological aspects of pelvic organ prolapse: A) fibrosis; B) congestion; C) inflammatory infiltrate; D) association of fibrosis, congestion and inflammatory infiltrate

**Morphological analyses:** concerning the morphological analyses, all slides were analyzed by an experienced pathologist blinded to the different groups during image capture and analysis. From each slide, 10 randomly selected fields using the Olympus CX23 microscope were captured at high magnification (200-400x). Fibrosis, inflammatory infiltrate, and blood vessels the size of engorged capillaries (congestion) were noted in each section or capture (Figure 1).

**Operational definitions:** fibrosis is the quantitative increase in macromolecules of the extracellular matrix [22]; the inflammatory infiltrate is composed of lymphocytes, plasma cells, and macrophages identified on captures from randomly selected fields [22]; congestion is the presence of engorged vessels the size of blood capillaries on captures from randomly selected fields [22].

**Statistical analyses:** we have analyzed our data using Statistical Package for Social Sciences (SPSS) software version 29. The ANOVA test was used to perform the intergroup comparison of means, and the Chi^2^ test to perform the intergroup comparison of proportions. The threshold of statistical significance of our results is set at the value of p < 0.05.

**Ethical considerations:** we respected the principles of medical ethics and documentary studies rules: data were collected confidentially and treated anonymously. Our study obtained authorization from the Ethics Committee of the Kinshasa Health School and the local committee of different hospitals. The reference number of the approval by the Ethics Committee is N°ESP/CE/19/2023. Each patient signed an informed consent form preoperatively, allowing the use of tissues removed during surgery for research purposes.

## Results

**General characteristics of the population:** the mean age of patients is 57.18 ± 8.17 years in the study group versus 56.48 ± 8.29 years in the comparison group and there are no statistically significant differences in the mean ages between the two groups. The mean parity of our patients is 7.76 ± 1.04 deliveries versus 2.76 ± 1.46 deliveries. Significant differences were noted between the mean parties in the two groups (Table 1).

**Table 1:** morphological anatomo-pathological aspects of pelvic organ prolapse

	Study group		Comparison group		Total		P-value
Age of patients: mean ± SD	57.18 ± 8.17		56.48 ± 8.29		56.83 ± 8.19		0.672
Parity: mean ± SD	7.76 ± 1.04		2.76 ± 1.46		4.76 ± 3.26		0.000
Isolated fibrosis	5	10%	8	16%	13	13%	0.280
Fibrosis + congestion	18	36%	23	46%	41	41%	
Isolated inflammatory infiltrate	4	8%	6	12%	10	10%	
fibrosis + inflammatory infiltrate	10	20%	6	12%	16	16%	
Fibrosis + congestion + inflammatory infiltrate	13	26%	6	12%	19	19%	
Fibrosis	46	92%	43	86%	89	89%	0.262
Congestion	31	62%	29	58%	60	60%	0.419
Inflammatory infiltrate	27	54%	20	40%	47	47%	0.115
Smooth muscles	11	22%	13	26%	24	24%	0.112

**Figure 2:**
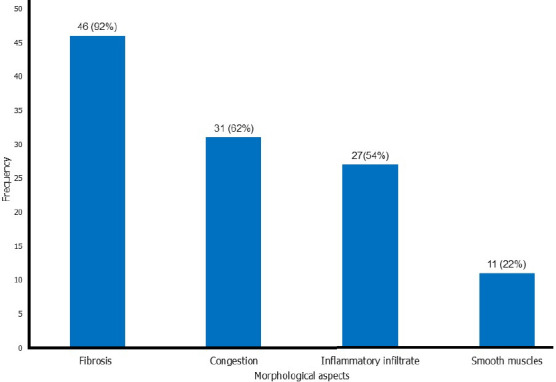
frequency of anatomo-pathological aspects of pelvic organ prolapse in the town of Kananga

**Anatomo-pathological aspects:** concerning the anatomopathological aspects, isolated fibrosis was noted in 5 cases of pelvic organ prolapse or 10%, fibrosis associated with congestion in 18 cases or 36%, inflammatory infiltrate composed of lymphocytes, plasma cells and macrophages in isolation in 4 cases or 8.00%, fibrosis associated with inflammatory infiltrate in 10 cases or 20% and fibrosis associated with congestion and inflammatory infiltrate in 13 cases or 26%. Overall, in the study group, fibrosis was present in 46 cases or 92%, congestion in 31 cases or 62%, and infiltrate of lymphocytes, plasma cells, and macrophages in 27 cases or 54% as well as smooth muscles in 11 cases or 22% (Figure 2). No statistically significant differences in the presence of smooth muscles (0.112), fibrosis (p = 0.262), congestion (p = 0.419), and inflammatory infiltrate (p = 0.115) were found between the study and comparison groups (Table 1).

## Discussion

The objective of our study is to determine the anatomo-pathological aspects associated with POP in round and utero-sacral ligaments of Congolese women registered during the surgical care campaign in two hospitals of Bon-Berger and Saint-Georges of the city of Kananga. Fibrosis was present in 46 cases or 92%, congestion in 31 cases or 62% and inflammatory infiltrate in 27 cases or 54%, and smooth muscle in 11 cases or 22%; and no statistically significant difference in the presence of fibrosis, congestion, and inflammatory infiltrate composed of lymphocytes, macrophages, and plasma cells were found between the study and comparison groups.

Fibrosis was present in 92% of cases of POP and its presence was not significant (p = 0.262), in both groups in our setting. This means that prolapse is not a fibrotic pathology. Our observations meet those of Kieserman-Shmokler *et al*. [21] and Ying *et al*. [29]. POP is characterized by the elevation of anti-fibrosis markers at the pelvic level, in particular, relaxin [30] and matrix metalloproteinases (MMPs) degrading collagen molecules including type I collagen in order to combat tissue fibrosis [20-27,31,32]. This confirms our results. Pelvic organ prolapse is also characterized by the presence of non-significant levels of tissue fibrosis markers such as fibroblast growth factor (FGF), transforming growth factor β (TGF-β), insulin-like growth factor (IGF), and the transmembrane glycoprotein CD44 whose role is to alter the balance between production and degradation of components of the extracellular matrix. This reflects the non-significant presence of fibrosis [29]. This explains our results.

Congestion was present in 31 cases of pelvic organ prolapse or 62% but its presence was not as significant (p = 0.419) in the 2 groups in our setting. This means that POP is not also a pathology of vascular congestion. Our results are consistent with those of Dviri *et al*. [22], Moalli *et al*. [26], Strinic *et al*.[24], Liang *et al*. [33] and Chen *et al*. [34]. This congestion can be explained by the way our biopsies were taken after clamping with two clamping forceps and the section between these two clamps but also by the frequent pelvic inflammations in our patients. Inflammatory infiltrate was present in 27 cases or 54.00% but its presence was also not significant (p = 0.115) in the ligament samples of both groups in our setting. This means that pelvic organ prolapse is not significantly an inflammatory pathology in our setting. Our findings are consistent with those of all authors who have worked on this subject in the literature in particular [20-27,31-33]. On the other hand, Wu *et al*. [35] and Li *et al*. [36], who examined the inflammatory pelvic environment in China, reported that patients with POP had a significantly higher inflammatory level in the tissues of the vaginal wall compared to the controls. This confirms that the important changes in the environment of pelvic inflammation are the basis of the pathogenesis of pelvic organ prolapse. Another study showed significantly elevated expression levels of matrix metalloproteinasis-2, cyclo-oxygenasis-2, and prostaglandin E2 in the patients with POP than in controls [37]. This means that inflammatory cytokines were released and expressed significantly in the vaginal wall of women with POP compared to controls: this is the basis of the alteration of collagen metabolism leading to POP. In addition, the interaction between oxidative stress and inflammation has now been proven and also aggravate or weaken the pelvic floor branch system [37]. Several studies have also found interactions between proinflammatory cytokines (interleukins 1-8) in fibroblasts and immune cells, and between those of inflammatory activators (interleukin-1B and interleukin-1R1) in pelvic smooth muscle cells [38]. These results simply mean that pelvic inflammation is involved in pelvic organ prolapse and that the microenvironment of inflammation could be an important factor in the occurrence of pelvic organ prolapse; nervertherless, enriching the understanding of these molecular biological mechanisms involved in the occurrence of POP requires additional studies in the future. These infiltrated inflammatory cells are responsible for the secretion of interleukins and tumor necrosis factor α (TNF-α) which stimulate the production of MMPs by fibroblasts and can also secrete MMPs whose role is the degradation of extracellular matrix components including collagen whose decreased content is significantly associated with pelvic organ prolapse [21-23,39,40].

Kieserman-Shmokler *et al*. in the United States of America reported the absence of significant differences in the presence of smooth muscles in the uterosacral and round ligaments of the study group compared to the comparison group [21]. This is also the case in our case. Dviri *et al*. in Israel [22], Strinic *et al*. in Croatia [24], Hu *et al*. in China [31], and Chen *et al*. in Taiwan [34] did not look for the presence of smooth muscles in the pelvic ligaments of their patients.

Our results imply the increased presence of inflammatory cytokines, fibrotic and anti-fibrotic markers associated with POP as matrix metalloproteinases, relaxin, interleukins… [35-38]; and can serve as a basis for further studies of assaying expression levels of matrix metalloproteinases associated with POP, inflammatory cytokines and relaxin in prolapsed pelvic connective tissues in our environment because the cells of the inflammatory infiltrate produce these interleukins, TNF-α and matrix metalloproteinases (MMP-1, -2, -9, ...) whose expression is significantly increased in patients with POP. Several studies today have demonstrated that interleukins and TNF-α activate the secretion, by fibroblasts, of matrix metalloproteinases which lead to the degradation of collagen, the reduction in pelvic content of which is significantly associated with the occurrence of POP [21-23,35-38,41].

Our study has the weakness of not having performed immunohistochemical assays of pelvic organ prolapse markers such as MMPs, inflammatory cytokines, and growth factors; and its strength is that it is the first to address the morphological anatomopathological aspects of POP in the hospital settings of the city of Kananga, the Democratic Republic of Congo and the African continent to our knowledge.

## Conclusion

Fibrosis, congestion, smooth muscle, and inflammatory infiltrate were present in the prolapsed round and uterosacral pelvic ligaments; and the comparison of patients with pelvic organ prolapse to those without prolapse did not find statistically significant differences between these two groups concerning the presence of fibrosis, congestion, the inflammatory infiltrate and smooth muscle. Our results can serve as a basis for experimental studies of dosage of expression levels of MMPs, inflammatory cytokines, and relaxin associated with POP in our environment since the cells of the inflammatory infiltrate produce interleukins, TNF-α which activate the production of matrix metalloproteinases (MMP-1, -2, -9, ...) which degrade collagen whose decrease is significantly associated with POP.

### What is known about this topic

- Pelvic organ prolapse (POP) is the fall of pelvic organs due to weakened pelvic support structures;
- Factors associated with POP in the city of Kananga are menopause, vaginal delivery malnutrition, multiparity, fetal macrosomia, obstetric trauma, and intense physical work;
- Data concerning the morphological anatomo-pathological aspects of POP in our milieu of Kananga do not exist.

### What this study adds

- Fibrosis, congestion, smooth muscle, and inflammatory infiltrate are present in the prolapsed round and uterosacral pelvic ligaments;
- There exists no significant difference in the presence of these anatomo-pathological aspects in patients with POP compared to those without prolapse;
- These results serve as a basis for studies of the dosage of expression of MMPs, inflammatory cytokines, and relaxin associated with POP in our city.
